# Utility of combined solid and liquid biopsy for molecular profiling in lung adenocarcinoma: Insights from a real-world case

**DOI:** 10.1016/j.jlb.2025.100303

**Published:** 2025-06-07

**Authors:** Bharat Bhosale, Gunj Bafna, Paridhy Vanniya Subramanyam, Kapil Salgia, Sadia Afreen, Jinumary John, Vyomesh Javle, Kritika Verma, N.R. Naavarasi, Sreekanth Reddy Peddagangannagari, Akhil Gorla, Giridharan Periyasamy, Kshitij Rishi, Hitesh Goswami, Vidya H. Veldore

**Affiliations:** a Bombay Hospital, Mumbai, India; b BB Precision Oncocare Centre, Mumbai, India; c SL Raheja Fortis Associate Hospital, Mumbai, India; d 4baseCare Precision Health Private Limited, Bengaluru, India

**Keywords:** Metastatic lung adenocarcinoma, Combined solid and liquid biopsy panel, Precision medicine, Crizotinib, Gefitinib

## Abstract

Lung adenocarcinoma is a subtype of NSCLC that is often associated with poor prognosis. We present a case of metastatic lung adenocarcinoma in which comprehensive genomic profiling using both solid and liquid biopsies was employed to monitor disease progression and guide targeted therapy decisions. An initial liquid biopsy detected *ROS1-CCDC6* gene fusion, and the patient was started on Crizotinib. Following disease progression, further genomic profiling using both solid tissue and cfDNA revealed the presence of a previously undetected classical mutation *EGFR* exon 21, p. L858R. Consequently, the treatment was adjusted to include both Crizotinib and Gefitinib. A 6-month follow-up showed relapse and extensive metastasis. A repeat liquid biopsy identified a newly acquired *TP53* mutation (exon 7, p.R248Q) in addition to the persistent *EGFR* mutation. Restarting the targeted therapy led to complete metabolic resolution of the disease. This case highlights the utility of liquid biopsy when tissue biopsy is not feasible and underscores the importance of integrating both solid and liquid genomic data to capture a more comprehensive mutational landscape, including low-frequency or emerging variants, ultimately enabling more effective, individualized treatment strategies.

## Highlights

• Liquid biopsy in a metastatic lung adenocarcinoma patient detected *ROS1-CCDC6* fusion; Crizotinib targeted therapy was given.
• Solid+liquid biopsy after disease progression detected *EGFR* p.L858R; modified therapy with Crizotinib+Gefitinib was started.
• Follow-up showed disease resolution, but new brain lesion was found. Targeted therapy was continued with radiation therapy.
• Disease progression with multiple metastases was seen in follow-up. Liquid biopsy detected *TP53* p.R248Q and *EGFR* p.L858R.
• Targeted therapy was restarted, resulting in complete metabolic resolution of primary and metastatic lesions.

Lung adenocarcinoma, a common subtype of non-small cell lung cancer (NSCLC), is associated with poor prognosis and is a major cause of cancer-related mortality (8.1%) in India [1]. The molecular pathogenesis of lung adenocarcinomas involves several mechanisms, with various oncogenic drivers that influence disease prognosis and disease management. Comprehensive genomic profiling (CGP), including liquid biopsy, has proven to be an invaluable tool in cancer management. It is estimated that 69% of advanced NSCLC patients have druggable targets [2], including well-known drivers- *Epidermal Growth Factor Receptor (EGFR), Anaplastic Lymphoma Kinase (ALK), ROS Proto-Oncogene 1, Receptor Tyrosine Kinase (ROS1), Kirsten Rat Sarcoma Virus Proto-Oncogene (KRAS), B-Raf Proto-Oncogene, Serine/Threonine Kinase (BRAF), Mesenchymal-Epithelial Transition factor Proto-Oncogene (MET)* and *Human Epidermal Growth Factor Receptor 2 (HER2)*. Identifying these therapeutic targets is crucial for efficient disease management and improving patient outcomes. Although liquid biopsy is not yet widely used in routine clinical oncology, ongoing research and optimization have demonstrated its efficacy, especially when tissue biopsy is not feasible. Besides being minimally invasive, liquid biopsy allows real-time monitoring of disease progression and provides insights into all tumor sites, unlike tissue biopsies where findings are limited to the specific tumor samples [3]. Here, we present a case of metastatic lung adenocarcinoma where liquid biopsy plays a key role in diagnosis, as well as in studying disease progression and monitoring.

A 57-year-old post-menopausal woman presented with loss of voice and cough for the past month, in May 2022. She neither had a history of any addictions or cancer in her family. An echocardiogram (ECHO) revealed pericardial effusion, and a thorax high-resolution computed tomography (HRCT) identified mediastinal lymphadenopathy with conglomerated necrotic nodes in the right paratracheal, subcarinal, precarinal, left paratracheal and aortopulmonary regions; the largest conglomeration in the subcarinal region measured 5.9 x 3.9 x 5.5 cm (transverse, TR x anteroposterior, AP x superior inferior, SI). Also, moderate pericardial effusion, a small nodular density measuring 9 mm in the left lung lower lobe, and patchy ground-glass opacities in the superior segment of right lower lobe and subpleural aspect of left lower lobe in lungs, were seen.

**Fig. 1:**
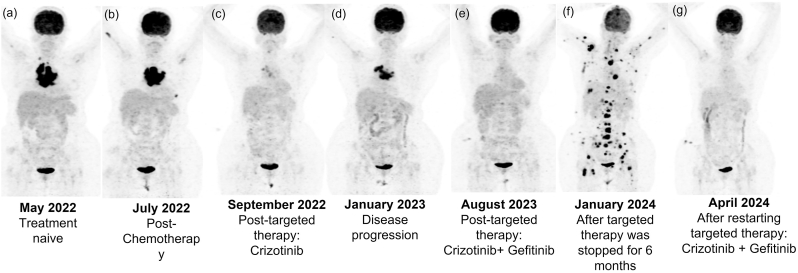
Series of PET-CT images of the patient at various time points throughout the course of treatment.

A computed tomography (CT)-guided core needle biopsy (CNB) of the subcarinal lymph node revealed metastatic high-grade carcinoma, with histopathological features suggesting thymic carcinoma. Testing for *Mycobacterium tuberculosis* was negative. A positron emission tomography (PET)-CT showed hypermetabolic, necrotic, and conglomerated mediastinal nodes and necrotic mildly enlarged right supraclavicular node, a small soft tissue density nodule in left lung lower lobe. Additionally, moderate pericardial effusion and focal low-grade metabolism was observed in the proximal shaft of right humerus, D4 vertebra and the left scapular spine (Fig. 1a). Reassessing the histopathology confirmed high-grade or poorly differentiated adenocarcinoma of pulmonary origin. The patient was treated with a cycle of chemotherapy (Paclitaxel (300 mg) and Carboplatin (450 mg)) in June 2022 (Fig. 1b), and comprehensive genomic profiling (CGP) was recommended. CGP with a large gene panel was attempted, but failed due to limited tissue availability. Therefore, cell-free DNA (cfDNA) was screened with large gene liquid biopsy panel, which detected the presence of *ROS Proto-Oncogene 1, Receptor Tyrosine Kinase- Coiled-Coil Domain Containing 6 (ROS1-CCDC6)* gene fusion. The molecular tumor board (MTB) recommended Crizotinib and Entrectinib for *ROS1* fusion-positive NSCLC; consequently, the patient was treated with Crizotinib (250 mg). In July 2022, the patient presented with cardiac tamponade and was shifted to intensive cardiac care unit where 200 ml of pericardial fluid was drained, which was further sent for cytology cell block preparation.

Response evaluation post-targeted therapy of 2 months with Crizotinib (250 mg) with PET-CT in September 2022 showed significant regression in the mediastinal and subcarinal lymph nodes, as well as marginal regression of the left lung lower lobe. Mild residual pericardial effusion and metabolic resolution of skeletal lesions were seen (Fig. 1c). However, a 5-months follow-up PET-CT scan, in January 2023, showed disease progression, with increased size and metabolism of the mediastinal and subcarinal nodes with infiltration of the left main bronchus. Further, previously left lung lower lobe nodule had increased in size and two new small semi-solid nodules were detected along with hypermetabolism in the right iliac bone and persistent reparative sclerosis in prior skeletal lesions (Fig. 1d).

A repeat CGP with a Solid-Liquid Combo large gene panel, which analyses both tumor tissue and plasma cfDNA was done. Since tissue biopsy was not feasible due to the proximity of major mediastinal vessels, cytology cell blocks previously prepared from pericardial fluid and fresh liquid biopsy were used for the CGP. Liquid biopsy detected the *EGFR* exon 21, p.L858R mutation and a *Myosin XVIIIB (MYO18B)* variant of uncertain significance (VUS: intron 36, c.5748+1G > A), with no trace of the earlier *ROS1* fusion. Analysing the retrospectively sent cytology block confirmed the *EGFR* mutation, which had been previously missed in the initial liquid biopsy. MTB recommended Food and Drug Administration (FDA)-approved drugs Osimertinib, Dacomitinib, Erlotinib, Afatinib, Gefitinib, for targeting the *EGFR* exon 21, p.L858R mutation. Due to financial constraints, the patient was treated with Crizotinib (250 mg) and Gefitinib (250 mg).

During a 6-month follow-up, the patient presented with headache and skin lesions. PET-CT showed complete metabolic resolution of the left lung lower lobe lesion and mediastinal/subcarinal lymphadenopathy. Two enhancing lesions in the cerebrum, suggesting metastasis, along with D4 and D12 vertebrae hypermetabolism was seen (Fig. 1e). Magnetic resonance imaging (MRI) of brain confirmed presence of two nodular lesions in the left parieto-occipital white matter with perilesional edema, consistent with brain metastases. The patient received stereotactic radiosurgery (SRS) on the brain lesions and tolerated the treatment well. A week later, the patient presented to the emergency room with high fever, chills, cough, breathlessness, fatigue, and hypoxia. HRCT chest showed bilateral pneumonia along with cardiac arrhythmias; the patient was placed on non-invasive ventilation. While initially bacterial pneumonia was suspected, *Mycobacterium tuberculosis* was detected on GeneXpert and culture. The patient was on AKT4 therapy (Isoniazid + Rifampicin + Ethambutol + Pyrazinamide) for 6 months for pulmonary tuberculosis; however, when faced with persistent arrhythmias due to drug interactions. Hence, targeted therapy with Crizotinib and Gefitinib was paused while the patient was closely monitored. A bronchoscopy and bronchoalveolar lavage showed no malignancy, at the time of discharge.

Later, 6-month follow-up PET-CT revealed disease progression. Thin-walled cavities in the left lung lower lobe and right lung upper lobe with fibrotic stranding, with mild left pleural effusion and hypermetabolic lesions were detected in the liver (bi-lobar), right adrenal gland, pancreas, uterus, and subcutaneous and intramuscular tissues, as well as increase in number of skeletal lesions. The previously identified left parieto-temporal lesion enlarged into a ring-enhancing mass, obliterating the left lateral ventricle and a new hypermetabolic left anterior periventricular lesion with perilesional edema was seen (Fig. 1f).

**Fig. 2:**
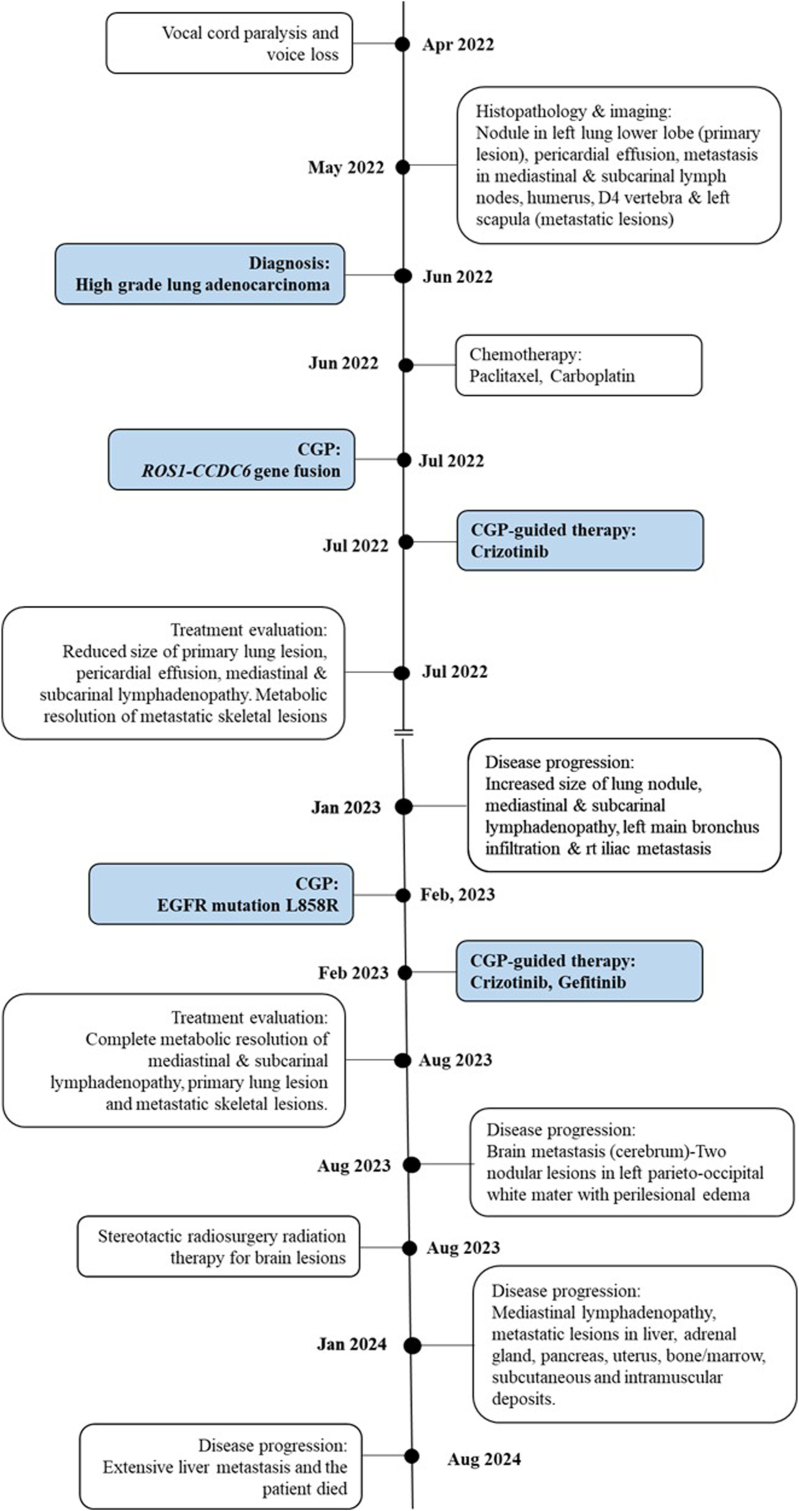
Comprehensive timeline of events in the case study.

Following this, the patient underwent another CGP with liquid biopsy using a small gene panel, which identified a *Tumor Protein P53 (TP53)* exon 7, p.R248Q mutation in addition to the previously detected *EGFR* exon 21, p.L858R mutation. Based on these findings, the patient resumed targeted therapy with Crizotinib and Gefitinib, showing a positive response and complete metabolic resolution of the metastatic and lung lesions (Fig. 1g). However, in August 2024, a blood profile showed elevated liver enzymes, and MRCP revealed extensive liver metastasis with hepatomegaly and retroperitoneal adenopathy. The patient was advised to receive symptomatic care but tragically passed away shortly thereafter in August 2024. A cohesive timeline of the case is summarized in Fig. 2.

In this case, the initial lymph node CNB did not yield sufficient quality DNA for next-generation sequencing (NGS), necessitating liquid biopsy. Given the inadequate tissue sample and the tumor's location near mediastinal vessels, liquid biopsy was a suitable non-invasive alternative. *ROS1* fusion was identified and the patient was started on Crizotinib, which has shown superior efficacy over platinum-based therapy in *ROS1*-positive NSCLC [4]. A study reported a 69% objective response rate (ORR) and 85% disease control rate (DCR) in *ROS1*-rearranged cases treated with Crizotinib [5].

Although the patient initially responded well, disease progression prompted a repeat CGP, which identified *EGFR* exon 21, p.L858R mutation, which also present in the retrospectively sent cell block and was missed in the first liquid biopsy. This classical *EGFR* mutation confers sensitivity to EGFR-TKIs and the patient was treated with Gefitinb, an FDA approved first-generation EGFR-TKI [6]. Later there was further disease progression with widespread metastases involving liver, adrenal glands, pancreas, uterus, bone/marrow, and soft tissues. About one-third of lung cancer patients develop extra-thoracic spread [7]. While metastases to the liver, adrenal glands, brain, and bone are common, pancreatic, subcutaneous, and intramuscular spread are rare. Bone metastases, especially in weight-bearing bones, require palliative management including analgesia, bone-targeted therapy, and local radiotherapy [8]. Soft tissue metastases from lung adenocarcinoma are rare (∼2.3%) and aggressive [9], with cutaneous metastasis indicating poor prognosis and a median survival of only a few months [10]. Another CGP identified after this identified a *TP53* mutation, known to be associated with poor outcomes. *TP53* mutations occur in ∼50% of NSCLC cases [11] and are linked to worse overall survival—27 months in wild-type versus 19 months in mutated cases(p < 0.001) [12]. According to recent European Society of Medical Oncology (ESMO) guidelines, *TP53* co-mutations may reduce the efficacy of targeted therapies (EGFR, ALK, ROS1 TKIs), underlining the importance of testing for these alterations. This case demonstrates the importance of CGP in identifying actionable mutations at different disease stages, highlighting its role in tracking tumor evolution and guiding treatment. A combined solid and liquid biopsy NGS approach (SoLiQ strategy) offers deeper insights into tumor dynamics, supporting more informed treatment decisions.

## Author contributions

BB, KS, GB, SA: Patient clinical management; PVS: Manuscript drafting; JJ, KV: Data analysis; VJ, NNR, SRT, AG: Bioinformatic data analysis; GP, VHV, BB: Critical revision of the manuscript; KR, HG: provided resources for the study and manuscript review; VHV, BB: Study conceptualization.

## Funding

This study was funded by In-house 4baseCare funding.

## Declaration of competing interest

The authors declare no conflict of interest.
